# Supplementary material for: Anti-resonant acoustic waveguides enabled tailorable Brillouin scattering on chip
Source: Nat Commun. 2024 May 8;15:3877. doi: 10.1038/s41467-024-48123-5 (PMC11078926; doi:10.1038/s41467-024-48123-5)
Supplement: Supplementary file 1 — Supplementary Information [file 41467_2024_48123_MOESM1_ESM.pdf]

Supplementary Information: Anti-resonant acoustic waveguides enabled tailorable Brillouin scattering on chip

Peng Lei[1], Mingyu Xu[1], Yunhui Bai[1], Zhangyuan Chen[1], and Xiaopeng Xie[1,†]

[1]State Key Laboratory of Advanced Optical Communication Systems and Networks, School of Electronics, Peking University, Beijing 100871, China

Corresponding authors: [†]xiaopeng.xie@pku.edu.cn.

CONTENTS

I. Linear and nonlinear loss model 1

II. Phase-matching and gain model of SBS 2
A. Phase-matching of SBS 2
B. Brillouin gain model 3

III. Anti-resonance condition and optimization using genetic algorithm 3
A. Anti-resonance condition 3
B. Optimization of SARAWs using genetic algorithms 5
C. Frequency selectivity of SARAWs 5

IV. Inductively coupled plasma etching process based on loading effect 5

V. Heterodyne four-wave mixing experiment 6

VI. Three-tone direct gain experiment 6

VII. The backward SBS experiment 8

VIII. Inhomogeneous broadening and peak splitting 8

References 10

I. LINEAR AND NONLINEAR LOSS MODEL

The optical linear and nonlinear losses are crucial parameters in the estimation of the Brillouin gain. The propagation loss of optical power (P) along z direction in silicon waveguides can be described by the following differential equation [1]

$$\frac{dP}{dz} = -\alpha P - \beta P^2 - \gamma P^3. \tag{S1}$$

Here, α is the linear loss coefficient. β and γ are the nonlinear loss coefficients of two-photon absorption (TPA) and TPA-induced free-carrier absorption, respectively.

The linear loss coefficient (α) of the fundamental TE mode in SARAWs can be obtained by measuring the optical quality factor of micro-rings with the corresponding central waveguide width. This method is insensitive to fluctuations of coupling losses. Considering the Fabry-Pérot (FP) background caused by the grating coupler reflection, the transmission spectrum of the micro-ring resonator is given by [2, 3]:

$$T_{total}(\Delta) = |t_1 t_2|^2 \frac{|T_{res}(\Delta)|^2}{|1 - r_1 r_2 T_{res}(\Delta)^2 e^{-i\Delta/\omega_{FP} + i\phi}|^2}, \tag{S2}$$

where t_1 (t_2) and r_1 (r_2) are the transmission and reflection coefficients of the grating couplers, respectively. T_{res} is the linear transmission function of the resonator. Δ is the detuning of the pump from the resonance frequency of the micro-rings. ω_{FP} is the free spectral range of the FP background, and ϕ is a phase offset. $T_{res} = 1 - \kappa_{ex}/(\kappa/2 + i\Delta)$, with the total resonator loss $\kappa = \kappa_{in} + \kappa_{ex}$, the intrinsic loss rate κ_{in}, and the external coupling rate κ_{ex}. By fitting the quality factor of the fabricated micro-ring resonators with different central waveguide widths W_1 (Fig.2h in the main text) through Eq.S2, we can obtain the intrinsic loss rate κ_{in}, and the derived intrinsic quality factors (Q_{in}) are 2.2×10^5, 4.0×10^5, and 8.5×10^5 (Supplementary Fig.1a-c), corresponding to W_1 = 450, 700, 1,200 nm separately. Utilizing the relation between Q_{in} and α [4],

$$Q_{in} = \frac{2\pi n_g}{\lambda\alpha}, \tag{S3}$$

with the group index n_g and the optical wavelength λ, the corresponding linear losses are 3.3, 1.6, and 0.3 dB/cm. A wider waveguide is less sensitive to sidewall roughness [5], resulting in lower α.

The nonlinear losses are mainly results from two-photon absorption and free-carrier absorption, and the nonlinear coefficients can be expressed as:

$$\beta = \frac{\beta_{TPA}}{A_{eff}},\ \gamma = \frac{\sigma\tau\beta_{TPA}}{2h\nu A_{eff}^2}, \tag{S4}$$

with the bulk TPA coefficient β_{TPA}, the effective mode areas A_{eff}, the free-carrier absorption cross-section σ, the free-carrier lifetime τ, and the energy of a single photon $h\nu$. Taking advantage of the lower linear loss characteristics of SARAW with a wider central waveguide width, we used SARAWs with a central waveguide width of 1,200 nm to achieve a large net gain and calculate their nonlinear loss coefficients. Through a full vectorial model [6], A_{eff} is calculated to be 1.674×10^{-13} m^2. Referring to $\beta_{TPA} = 7.9\times10^{-12}$ mW^{-1} and $\sigma = 1.45\times10^{-21}$ m^2 in bulk silicon [7], β is 47 m^{-1}W^{-1}. By deploying the

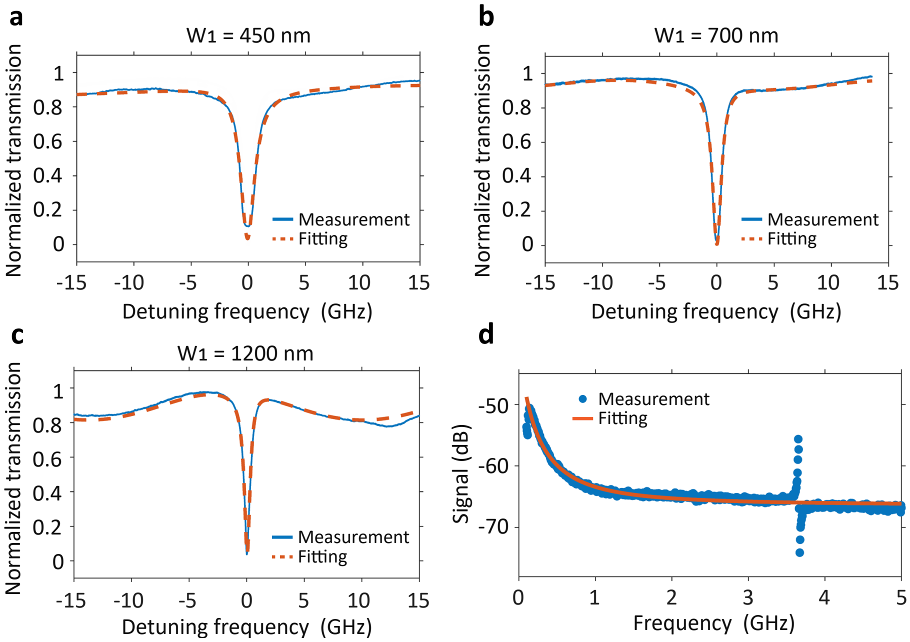

Supplementary Fig. 1. Measurement of linear and nonlinear loss. a-c The optical quality factor fittings with FP background. **d** The measurement of free carrier lifetime using heterodyne FWM experiment. The Brillouin resonance is observed near 3.6 GHz, corresponding to the frequency of Brillouin resonance in Fig.3c in the main text.

free-carrier lifetime measurement based on heterodyne four-wave mixing (FWM) experiment [1], the fitted τ is 2.6 ns (Supplementary Fig.1d), and the calculated γ is 4,150 $\mathrm{m}^{-1}\mathrm{W}^{-2}$.

II. PHASE-MATCHING AND GAIN MODEL OF SBS

A. Phase-matching of SBS

The process of SBS demands the conservation of energy and momentum, imposing strict phase-matching requirements among the pump, Stokes, and acoustic waves. Depending on the relative propagation directions of the pump and Stokes lights, intramodal SBS can be classified into forward SBS (FSBS) and backward SBS (BSBS), each corresponding to acoustic modes with distinct characteristics. The dispersion relations of the optical and acoustic modes for forward and backward SBS are shown Supplementary Fig.2a,b, respectively.

In forward SBS, the Brillouin nonlinearity occurs between the co-propagating optical waves. The pump light (blue circle) is coupled to the Stokes light (red circle) and generates a FSBS acoustic phonon with a frequency of $f_{fa} = \nu_p - \nu_{fs}$ and a wave-vector of $q_f = k_p - k_{fs}$ (Supplementary Fig.2a). Here, ν_i, k_i ($i = p,\ fs$) are the frequencies and wave-vectors of the pump wave (p), and the forward Stokes wave (fs). Due to the significantly slower acoustic velocity in comparison to optical velocity, the wave-vector of the acoustic wave in FSBS, denoted as q_f, is extremely small (near zero). And the group velocity v_g, equivalent to the slope of the tangent line to the dispersion curve (gray dashed lines in Supplementary Fig.2b), is generally slower for FSBS.

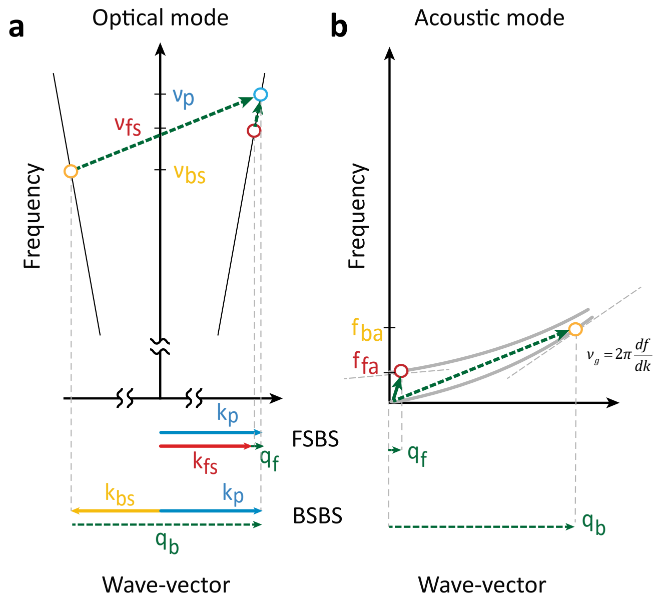

Supplementary Fig. 2. Dispersion relations. a Energy conservation and phase-matching for forward and backward SBS. **b** Dispersion relations of acoustic modes for forward and backward SBS.

In backward SBS, the Brillouin nonlinearity occurs between the counter-propagating pump wave (blue circle) and Stokes wave (yellow circle), as illustrated in Sup-

plementary Fig.2a. The resulting phonon wave-vector is $q_b = k_p + k_{bs}$, almost twice as large as k_p. It is much larger than q_f. Therefore, to satisfy the phase-matching requirement of BSBS, the acoustic mode generally has a higher eigenfrequency, as shown in Supplementary Fig.2b. Additionally, the backward acoustic mode exhibits a larger group velocity compared to FSBS. It implies that, with comparable phonon lifetimes, BSBS phonons exhibit significantly longer propagation distances compared to FSBS phonons.

B. Brillouin gain model

For pump power (P_p) and Stokes power (P_s), the coupled differential equations along the propagation direction (z-axis) with linear and nonlinear losses are [1]:

$$\frac{1}{P_p(z)}\frac{dP_p(z)}{dz} = -G_B(\Omega)P_s(z) - \alpha - \beta P_p(z) - \gamma P_p(z)^2, \tag{S5}$$

$$\pm\frac{1}{P_s(z)}\frac{dP_s(z)}{dz} = G_B(\Omega)P_p(z) - \alpha - 2\beta P_p(z) - \gamma P_p(z)^2, \tag{S6}$$

where the $\pm$ corresponds to forward and backward SBS, respectively. $G_B(\Omega)$ is the Brillouin gain coefficient and can be expressed as [8]:

$$G_B(\Omega) = \frac{2\omega_p Q_m L(\Omega)}{m_{eff}\Omega_a^2}|\int f_{ES}dA + \int f_{RP}dl|^2, \tag{S7}$$

where ω_p is the angular frequency of the pump light. Q_m and Ω_a are respectively the mechanical quality factor and the angular eigenfrequency of the acoustic mode. $L(\Omega) = (\Gamma/2)^2/[(\Gamma/2)^2+(\Omega-\Omega_a)^2]$ is the Lorentzian gain profile for an acoustic mode with the acoustic damping rate Γ. $m_{eff} = \int \rho|\mathbf{u}|^2/max|\mathbf{u}|^2 dA$ is the effective mass of an acoustic mode with displacement field $\mathbf{u}$ and density ρ. The last two items are the area overlap integrals of the electrostrictive force f_{ES} and line overlap integrals of the radiation pressure f_{RP}, respectively. Dividing Q_m to the left side of Eq.S7 yields the coupling factor G_B/Q_m, which reflects the overlap integral of optical and acoustic modes. The simulations and optimizations of the coupling factor in Methods are based on this expression.

III. ANTI-RESONANCE CONDITION AND OPTIMIZATION USING GENETIC ALGORITHM

A. Anti-resonance condition

The acoustic modes in waveguides can be obtained by solving the elastic equation of motion [9]

$$\rho\frac{d^2\mathbf{u}}{dt^2} = \nabla\cdot\mathbf{T}, \tag{S8}$$

where ρ is the density, $\mathbf{u}$ is the displacement field and $\mathbf{T}$ is the stress tensor. For an ideal planar waveguide (upper part of Fig.1a in the main text), the entire structure can be decomposed into layers with different acoustic velocities. Eq.S8 can be applied in each layer and solved numerically with appropriate boundary conditions. Analogous to an optical Fabry-Pérot cavity, under ideal conditions, the acoustic resonance and anti-resonance conditions can be approximately expressed as [9]:

$$k_s t = n\pi, n \in \mathbb{Z}^+, \tag{S9}$$

$$k_s t = (n-0.5)\pi, n \in \mathbb{Z}^+, \tag{S10}$$

respectively, where k_s is the transverse wave-vector. Acoustic resonance and anti-resonance states appear periodically and alternately, as slot width t increases. Under the anti-resonance states, the acoustic waves in the slow-velocity layers experience destructive interference, confining the acoustic field within the central layer. On the other hand, the acoustic waves are primarily distributed in the slow-velocity layers under the resonance states.

Due to complex boundary conditions and structural distortions of the etched slots, the resonance and anti-resonance conditions of SARAWs can not be directly solved using Eq.S9. Therefore, we employ the finite element solver COMSOL to numerically solve the acoustic frequencies and acoustic mode distributions under various geometric structures.

In order to further illustrate the resonance and anti-resonance conditions of SARAWs and demonstrate the corresponding acoustic mode distributions, we set the waveguide width W to 700 nm and scanned the slot width t up to 2 μm (Supplementary Fig.3a). To evaluate the acoustic energy confinement effect under different parameters, we tracked normalized energy, defined as the ratio of acoustic mode energy in the central waveguide to the total acoustic mode energy, as shown in Supplementary Fig.3b. And we performed scans for two cases with the remaining thickness of the silicon layer T = 15 nm (blue lines) and 30 nm (orange lines). The bottom boxes of Supplementary Fig.3b display two types of acoustic modes of the etched slots under resonance conditions, where the left mode (blue box) can be interpreted as a shear mode along the slot in the horizontal direction, and the right mode (red box) can be interpreted as a longitudinal mode along the slot in the horizontal direction. To realize acoustic anti-resonance in SARAWs, it has to meet the anti-resonance conditions of the shear and longitudinal modes simultaneously.

As slot width t increases (Supplementary Fig.3b), the variation of the normalized energy is a superposition of two distinct periodic oscillation profiles. For the case of the remaining thickness of the silicon layer T = 15 nm, the narrower dips of the normalized energy curve correspond to the resonance conditions of the shear mode, with a repetition period of approximately 100 nm. Meanwhile, the wider dips correspond to the resonance condi-

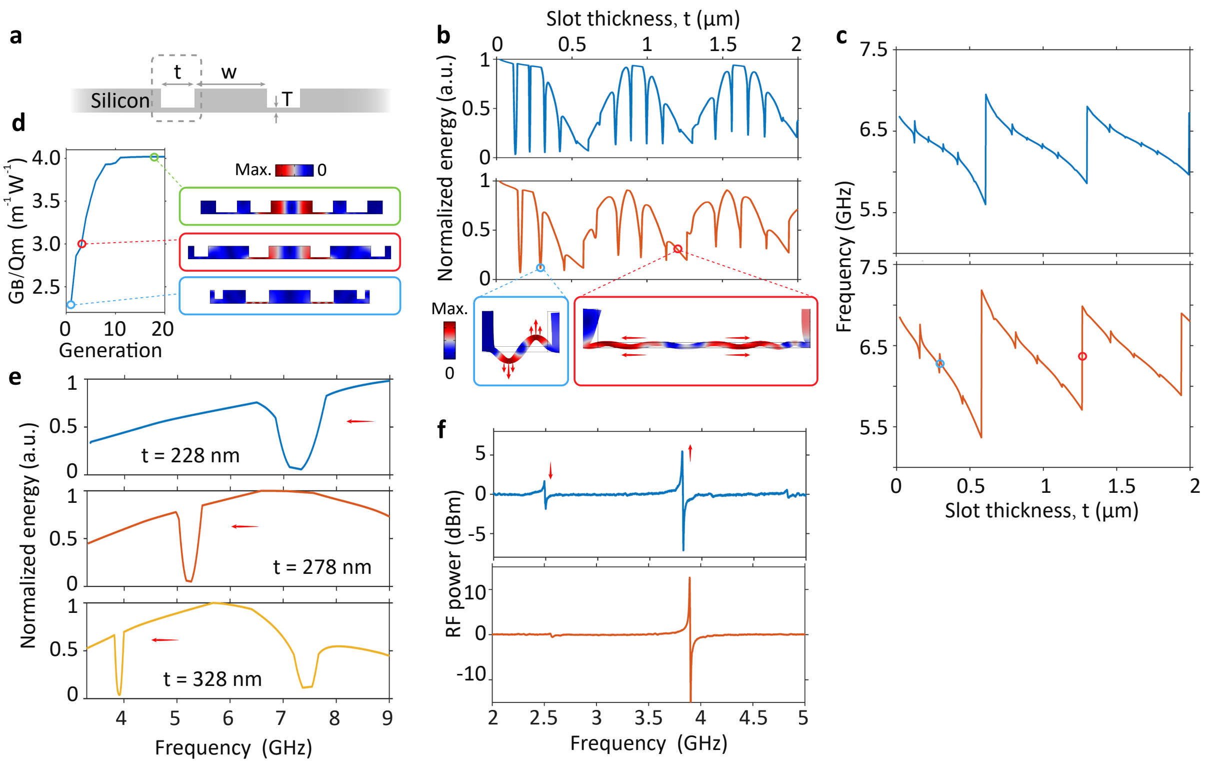

Supplementary Fig. 3. Characteristics of acoustic anti-resonance. **a** Schematic of etched slots on a silicon membrane. **b** The relationship between the slot width and the normalized acoustic mode energy in the waveguide. The deformation in the blue and red boxes is magnified proportionally according to the displacement field. **c** The relationship between the slot width and the eigenfrequency of the Brillouin-active mode. The blue and red circles on the orange line show the resonance condition for shear mode and longitudinal mode, corresponding to the acoustic modes marked by the circles and boxes on the orange line in figure **b**. In figure **b,c**, the blue and orange lines correspond to the remaining silicon layer thickness T = 15 and 30nm, respectively. **d** The optimization process of the genetic algorithm and the corresponding variation of the elastic displacement field. **e** Normalized acoustic energy with varied acoustic frequency. Three cases with slot width t = 228, 278, 328 nm are shown. **f** Illustration of frequency selectivity in SARAWs. The top is the heterodyne FWM measurement result before optimization; the bottom is the heterodyne FWM measurement result for SARAWs with enhanced phonon mode near 4 GHz.

tions of the longitudinal mode, with a repetition period of approximately 700 nm. Compared to the case of T = 30 nm, the shear mode exhibits a notable relative change in the repetition period, increasing from 100 nm to 150 nm. On the other hand, the longitudinal mode experiences a slight shift in the repetition period, decreasing from 700 nm to 670 nm. This disparity is primarily attributed to the pronounced influence of the remaining silicon layer thickness T on the shear wave velocity. In contrast, the longitudinal wave velocity remains relatively constant, resulting in the stable resonance conditions of the longitudinal mode. Since the eigenfrequency of Brillouin-active acoustic mode in the central waveguide with waveguide width W = 700 nm is approximately 5.9 GHz, the velocities of the shear mode in the etched slots are 1755 and 1170 m/s, corresponding to T = 30 and 15 nm respectively. The fact, that the velocity in etched slots decreases as the T decreases, enables etched slots to form equivalent slow velocity layers for anti-resonant reflecting. It acts as the basic principle for constructing anti-resonant structures in SARAWs. The uneven appearance of resonance conditions in Fig.2b-d in the main text also originates from this. As SARAWs are fabricated using the loading-effect-based technique, wider slot width results in smaller T, causing the resonance and anti-resonance conditions to drift.

Supplementary Fig.3c illustrates the frequency of the Brillouin-active acoustic mode as a function of the slot width t. When the resonance conditions for shear or longitudinal modes are met, the Brillouin-active acoustic mode in the central waveguide and the acoustic modes (shear or longitudinal) in the etched slots are strongly coupled. The phenomenon of avoided mode crossing is observed in both figures [10].

For structural stability, we have set the slot width (t) close to 250 nm, aligning with the first anti-resonance point of the longitudinal mode. The overall trend of the acoustic frequency and the coupling factor (G_B/Q_m) is only slightly affected by the longitudinal mode with increasing slot width (t) (Fig.2b-d in the main text). Consequently, we only focus on the anti-resonance condition of the shear mode in the main text.

B. Optimization of SARAWs using genetic algorithms

Considering the influence of the loading effect during the etching process, the theoretical prediction of anti-resonance conditions becomes complex. Additionally, obtaining the optimal structural parameters through parameter scans is time-consuming and impractical. Therefore, we employ the genetic algorithm to streamline the optimization process. We take the coupling factor (G_B/Q_m) as the fitness function for optimization. Because achieving the maximum coupling factor requires SARAWs to simultaneously confine both acoustic and optical modes, genetic algorithms will automatically optimize geometric parameters to satisfy the anti-resonance conditions of the acoustic mode.

As an example, Supplementary Fig.3d illustrates the optimization process for SARAWs with $W_1 = 700$ nm. The genetic algorithm converges after 10 generations, and the acoustic displacement field is squeezed into the central waveguide (green box), corresponding to the optimal coupling factor. The optimized parameters (W_1, W_2, W_3, t_1, t_2, t_3, d_1, d_2, d_3 (unit nm)) corresponding to Fig.2h in the main text are (450, 138, 189, 242, 266, 1,500, 194, 196, 220), (700, 223, 255, 346, 356, 1,500, 193, 194, 220), and (1,200, 394, 402, 500, 419, 1,500, 200, 197, 220), respectively. The parameters of the SARAW with $\mathrm{W}_1 = 700$ nm in Fig.1,2 in the main text are also derived from here.

We emphasize that the genetic algorithm offers a rapid approach to optimize targeted parameters. By designing the fitness function appropriately, we can optimize not only the coupling factor, but also the mechanical quality factor and mode field profile for specific acoustic modes based on the application scenario.

C. Frequency selectivity of SARAWs

To further illustrate the acoustic frequency selectivity of SARAWs, we swept the central waveguide width W (Supplementary Fig.3a) to obtain varied eigenfrequencies of the Brillouin-active acoustic mode. By controlling W to scan the acoustic frequency from 4 to 9 GHz (Supplementary Fig.3e), we tracked the variation of the normalized acoustic mode energy of the central waveguide for different slot widths t with the remaining silicon layer thickness T of 30 nm. The blue, orange, and yellow curves represent the cases of slot width t = 228, 278, and 328 nm, respectively. Clear peaks and dips appear in the curves, corresponding to the anti-resonance and resonance frequencies with the given slot width. Anti-resonant peaks serve to enhance the acoustic energy, while resonant dips act as inhibitors, suppressing the acoustic energy. Leveraging this characteristic, we can selectively filter or suppress acoustic modes at specific frequencies. As the slot width (t) increases, both the resonance and anti-resonance frequencies shift to lower values, and a second resonance frequency emerges when t = 328 nm. This resembles the dependence of the free spectral range (FSR) on the length of the Fabry-Pérot cavity, and enables us to manipulate the frequency selectivity by adjusting the anti-resonant structures.

Utilizing this frequency selectivity, SARAWs can selectively enhance a specific acoustic mode while suppressing others. For a particular SARAW of $W_1 = 1{,}200$ nm, it can support multiple acoustic modes, resulting in the appearance of multiple peaks in the measurement result of the heterodyne FWM experiment (the top of Supplementary Fig.3f). By adjusting the anti-resonant structures, the SARAW can suppress the 2.5 GHz mode and enhance the acoustic mode near 4 GHz (the bottom of Supplementary Fig.3f). Leveraging the property of selectively enhancing a specific acoustic mode and suppressing others, SARAWs offer a more versatile design dimension.

IV. INDUCTIVELY COUPLED PLASMA ETCHING PROCESS BASED ON LOADING EFFECT

Loading effect is an etching phenomenon in which the etching rate depends on the aperture size due to the consumption of reactants, as illustrated in Supplementary Fig.4a. The etching rate increases with wider aperture width. This effect is also referred to as reactive ion etching (RIE) lag or aspect ratio dependent etching (ARDE) [11]. In this work, we fabricated a series of slots with varying widths and measured the etching depths to obtain the loading effect curves shown in Supplementary Fig.4b. The slot width in the anti-resonant structure (t_1 and t_2 in Fig.1c in the main text) is typically 300 $\sim$ 600 nm, resulting in the etching depth of 170 $\sim$ 200 nm, which prevents over-etching and ensures a stable connection for SARAWs. We emphasize that this curve (Supplementary Fig.4b) can be adjusted by tuning the inductively coupled plasma (ICP) recipe parameters, such as reactant species and ICP power. The curve exhibited good reproducibility. We incorporated it into the parameter constraints in the genetic algorithm, and achieved excellent agreement between the fabricated and simulated parameters.

Based on the loading effect, the fabrication process of SARAWs is depicted in Supplementary Fig.4c. First, a positive electron beam resist is spin-coated, followed by electron beam lithography (EBL) to transfer the designed pattern. After development, the pattern is etched by ICP. Due to the loading effect, the wider slots at the edges (t_3 in Fig.1c in the main text) are overetched to expose the underlying silica, which is then removed by wet etching with 10% hydrofluoric acid solution, thus achieving full suspension of the SARAW. With this fabrication process, only one exposure and etching step is required, eliminating the need for overlay exposure. It leads to very high fabrication precision for SARAW. According to our fabrication results, a sub-5 nm feature pitch can be achieved.

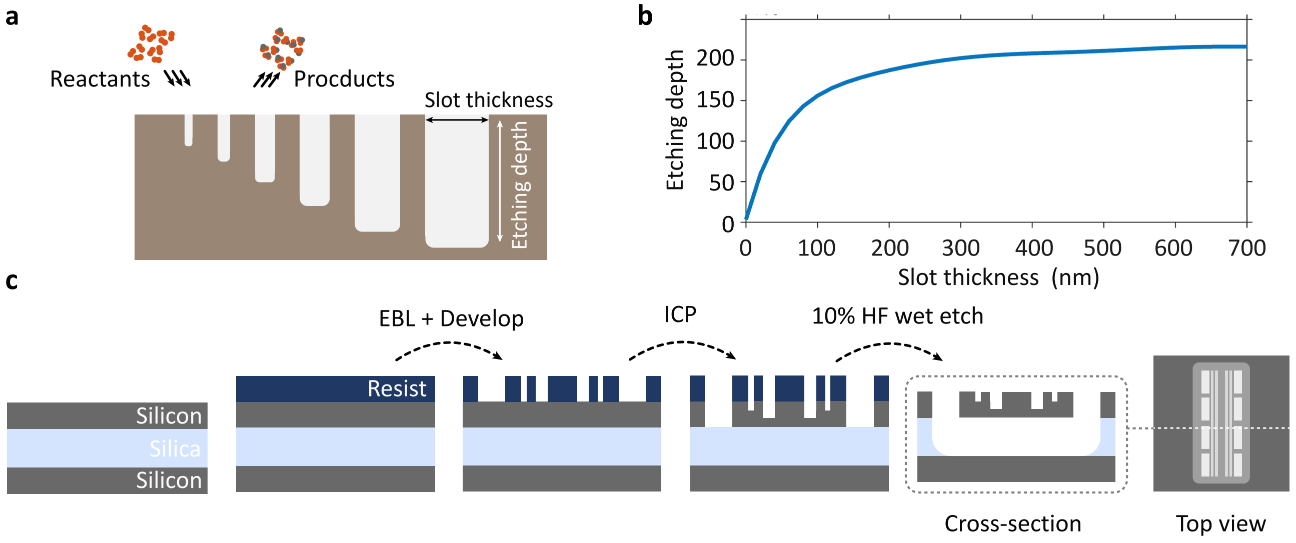

Supplementary Fig. 4. Inductively coupled plasma etching based on loading effect. **a** Diagram of loading effect. **b** Experiment and fitting of loading effects. **c** Fabrication process.

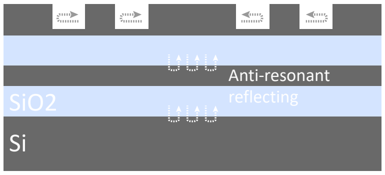

Supplementary Fig. 5. Anti-resonant reflection in the vertical direction

It should be noted that, for SARAWs, to prevent acoustic wave leakage into the silica substrate, a 10% HF under-etching step is employed to achieve waveguide suspension. It adopts an anti-resonant design only in the horizontal direction. However, we propose that growing silica-silicon layers vertically to establish anti-resonant reflecting layers could also be effective, as illustrated in Supplementary Fig.5. This design might eliminate the need for the under-etching step. Additionally, for materials like chalcogenide glass or aluminum nitride, which have acoustic velocities smaller than the silica substrate, acoustic waves are unable to leak downward. The SARAW structure can be seamlessly transplanted onto these platforms.

V. HETERODYNE FOUR-WAVE MIXING EXPERIMENT

The spectral shape of the heterodyne four-wave mixing measurement (Fig.2a in the main text) arises from the interference of the resonant Brillouin scattering and the background Kerr nonlinearity. It follows a Fano-like line shape given by [1, 12]:

$$f_L(\Omega) = \left| e^{i\varphi} + \frac{G_B L_{SBS}}{4\gamma_k L_{tot}} \frac{\Omega_a/(2Q_m)}{\Omega_a - \Omega - i\Omega_a/(2Q_m)} \right|^2, \tag{S11}$$

where φ is a relative phase between the Brillouin and Kerr nonlinearities, L_{SBS}/L_{tot} is the ratio of Brillouin active length to total waveguide length, and γ_k is the Kerr nonlinear coefficient. Based on the full vectorial model from RefS.[13], we obtain the γ_k values of SARAWs with waveguide widths $W_1 = 450$, 700, and 1,200 nm in Fig.2h in the main text, which are 257, 179, and 110 $W^{-1}m^{-1}$, respectively.

Applying Eq.S11 to fit the experimental results of heterodyne FWM measurement, we can derive the values of G_B and Q_m. For the three waveguide widths in Fig.2h in the main text, the fitting results of the Stokes and anti-Stokes spectra are shown in FigS6a-c. The corresponding values of G_B ($W^{-1}m^{-1}$) and Q_m are (1,800, 210); (3,530, 680); (1,860, 850), for waveguide width W_1 = 450, 700, and 1,200 nm, respectively.

VI. THREE-TONE DIRECT GAIN EXPERIMENT

The three-tone gain experiment [12] has a simpler setup than the traditional small-signal gain experiment. The setup in this work is shown in Supplementary Fig.7. The C.W. laser (frequency ν_1, wavelength 1,550 nm) is divided into two paths via a coupler. The upper branch passes through an IM and generates two sidebands ($\nu_1 \pm f_m$). The carrier acts as the pump light, and the sidebands act as the probe lights. To satisfy the small-signal condition, the sideband powers are set to be 25 dB lower than the carrier. The light in the bot-

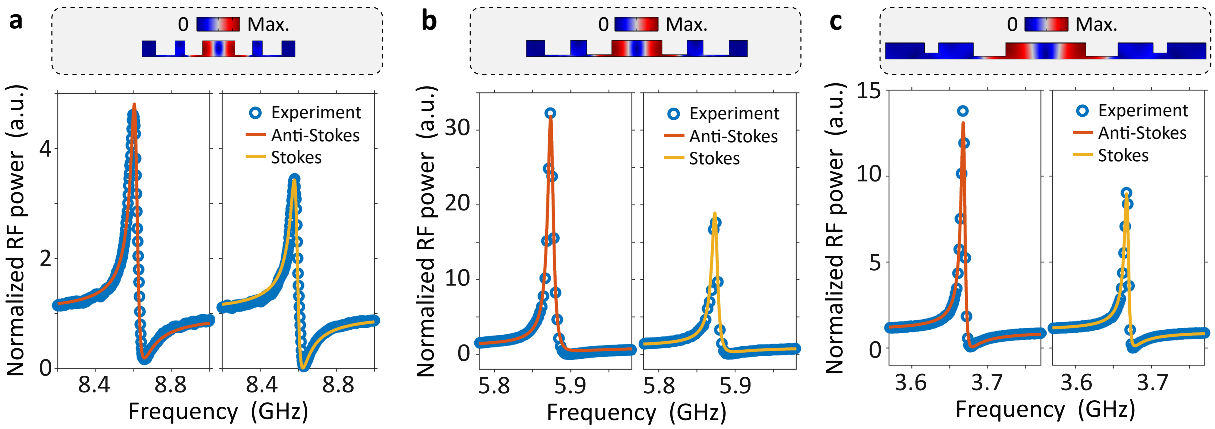

Supplementary Fig. 6. Simulation and experimental results of the heterodyne four-wave mixing experiment. a-c The elastic displacement magnitude (upper) and the fittings of the experimental results for optimized SARAWs are depicted for waveguide widths W_1 = 450, 700, and 1,200 nm, respectively.

tom path experiences a frequency shift to $\nu_1 + \Delta f$ via an AOM, and acts as a reference signal. In the DUT, SBS facilitates energy transfer between the pump and the two sidebands when f_m matches the Brillouin frequency shift. The strength of the energy transfer is directly related to G_B. After passing through the DUT, the light $(\nu_1 \pm f_m)$ is then combined with the reference signal $(\nu_1 + \Delta f)$ in the PD. The intensity variation of the beat signal $f_m + \Delta f$ is recorded by VNA, corresponding to the optical Stokes sideband $\nu_1 - f_m$.

In this process, the coupled amplitude equations for the pump (a_p), Stokes (a_s), anti-Stokes (a_{as}) and acoustic (a_a) waves can be expressed as [12]

$$\begin{aligned}
\frac{da_s}{dz} &= \frac{G_B\Gamma}{4}\chi^* a_p a_a^* - \frac{1}{2}(\alpha + 2\beta\,|a_p|^2 + \gamma\,|a_p|^4)a_s,\\
\frac{da_p}{dz} &= -\frac{G_B\Gamma}{4}(\chi a_s a_a - \chi^* a_{as} a_a^*)\\
&\quad - \frac{1}{2}(\alpha + \beta\,|a_p|^2 + \gamma\,|a_p|^4)a_p,\\
\frac{da_{as}}{dz} &= -\frac{G_B\Gamma}{4}\chi a_p a_a - \frac{1}{2}(\alpha + 2\beta\,|a_p|^2 + \gamma\,|a_p|^4)a_{as},\\
a_a &= a_s^* a_p + a_p^* a_{as}. \qquad \text{(S12)}
\end{aligned}$$

Here, the frequency response $\chi = 1/(\Gamma/2 + i(\Omega_a - \Omega))$. The optical powers $P_i = |a_i|^2$ (i = s, p, as).

In traditional small-signal gain experiment, only a single Stokes light is involved, and the small-signal gain is defined as the amplification of the probe light. In contrast, the three-tone gain experiment utilizes the coherent excitation of the acoustic field by both sidebands $(\nu_1 \pm f_m)$ [12]. This leads to a higher Stokes gain for the sideband $(\nu_1 - f_m)$ compared to the small-signal gain. Therefore, the three-tone experiment is more effective for detecting the smaller Stokes gain. However, to derive the corresponding traditional small-signal gain, we need to numerically solve Eq.S12 and fit the experimental data from three-tone experiment (Fig.3b in the main text). The obtained parameters are used to determine the small-signal gain using Eq.S5-6 (Fig.3d in the main text). Moreover, it should be noted that the mechanical quality factor Q_m can not be directly determined by the full width at half maximum (FWHM) of Fig.3a in the main text. Instead, it should be derived by numerically solving Eq.S12 and fitting the experimental results.

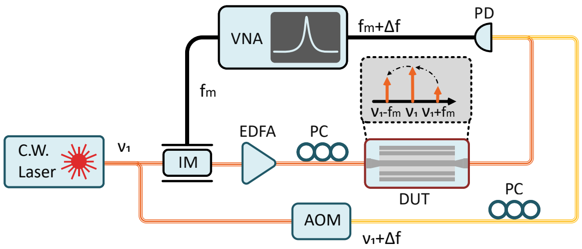

Supplementary Fig. 7. The setup of three-tone direct gain experiment. Abbreviations: C.W. Laser, continuous-wave lasers; IM, intensity modulator; VNA, vector network analyzer; EDFA, erbium-doped fiber amplifier; DUT, device under test; AOM, acousto-optic modulator; PD, photodetector; PC, polarization controller.

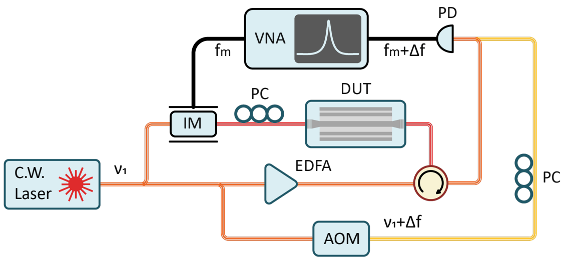

Supplementary Fig. 8. The setup of the backward SBS experiment.

TableS. 1. Comparison of Brillouin nonlinearity in integrated silicon waveguides.

Ref	Year	Length (cm)	Optical loss (dB/cm)	Scattering process	Brillouin frequency (GHz)	Mechanical quality factor	Gain coefficient ($W^{-1}m^{-1}$)	Net gain (dB)
[14]	2013	0.33	7	FSBS	1.8-16.3	1,000-1,800	2,750	-1.9
[15]	2015	4	0.18	FSBS	9.2	306	3,218	-0.1
				BSBS	13.7	971	357	-
[16]	2015	2.5	5.5	FSBS	9.1	728	6,561	0.5
[1]	2016	2.9	0.18	FSBS	4.4	680	1,152	5.2
[12]	2021	1.1	2	FSBS	4.45	566	1054	-1
This work	2024	2.5	0.3	FSBS	3.6-8.6	650-850	1,600-3,530	6.4
				BSBS	18.1-27.6	1,200-1,960	430-600	-

VII. THE BACKWARD SBS EXPERIMENT

SARAWs also have the capability to support backward Brillouin scattering. This arises from the fact that, even though the main component of the backward SBS acoustic wave is longitudinal, its wave-vector still possesses a component in the transverse direction, leading to the corresponding anti-resonance condition [9].

The setup of the backward SBS experiment is shown in Supplementary Fig.8. The light from a C.W. laser (frequency ν_1, wavelength 1,550 nm) is split into three paths. The light in the upper path passes through an IM and generates a sideband at $\nu_1 - f_m$ (25 dB below the carrier to meet small-signal condition), serving as the probe light. The light in the middle path is amplified by an EDFA and acts as the pump light, entering the DUT from the right through a circulator. When f_m matches the backward Brillouin frequency shift, the probe light undergoes amplification through the SBS process. After passing through the DUT, the probe light ($\nu_1 - f_m$) is further mixed with a reference signal at $\nu_1 + \Delta f$ from an AOM in the lower path branch. The beat signal ($f_m + \Delta f$) is measured by the VNA, and its intensity corresponds to the small-signal gain of backward SBS.

To obtain the G_B of backward SBS, we swept the pump power and measured the corresponding small-signal gain. Solving the Eq.S6 numerically and fitting the experimental data, the obtained G_B (Supplementary Fig.9a-d) are 530, 600, 430, and 480 $W^{-1}m^{-1}$, corresponding to Fig.4a-d in the main text respectively.

According to the phase-matching relationship (Supplementary Fig.2a), the group velocity of the acoustic mode, can be inferred by the slope of the tangent line to the acoustic dispersion curve. The acoustic modes depicted in Supplementary Fig.9a-d are similar to those in a suspended rectangular waveguide. Therefore, for the sake of simulation convenience, we simulate the acoustic dispersion of the suspended rectangular waveguide (220 nm $\times$ 450 nm) near a wave-vector of $2k_p$, as shown in Supplementary Fig.9e. The 18 and 28 GHz acoustic modes are consistent with the backward SBS acoustic modes observed in SARAWs. Their group velocities are 3,190 and 6,980 m/s, respectively. With a Q_m of 1,960 in Fig.4d in the main text, we estimate the acoustic decay length of the bell-shaped mode to be larger than 50 μm. The acoustic mode at the bottom of the gray dashed box in Supplementary Fig.9 corresponds to the Fabry-Pérot acoustic mode of backward SBS in nano wire on pillar [15]. Owing to its small G_B/Q_m, this mode is not the focal point of our investigation.

Referring to RefS.[17], we further compared our results of backward and forward SBS in SARAWs with recent studies conducted on silicon waveguides [1, 12, 14–16]. As shown in TableS.1, SARAWs have not only lower optical losses but also significantly higher mechanical quality factors and Brillouin gain coefficients, supporting a net gain of up to 6.4 dB. Moreover, we achieved the largest Brillouin frequency and mechanical quality factor to date through backward SBS in SARAWs. The design of SARAWs allows flexible adjustments for these metrics. We believe that the structural and design methodology outlined in this paper has the potential to usher in comprehensive breakthroughs for integrated Brillouin waveguides.

VIII. INHOMOGENEOUS BROADENING AND PEAK SPLITTING

The inhomogeneous broadening of Brillouin resonance caused by fabrication defects can be classified according to the spatial scale of the defects [18]. Defects with short-scale variations (compared with the acoustic decay length), such as sidewall roughness, may result in large acoustic energy dissipation and decline in the mechanical quality factor. On the other hand, defects with long-scale variations, such as non-uniformity of silicon membrane thickness (Supplementary Fig.10a), can induce drifts in the geometric parameters of the waveguide cross-section, thereby causing shifts in the acoustic frequencies. In extreme cases, it will result in peak splitting of Brillouin resonance, inducing a dramatic decrease in Brillouin gain.

Short-scale defects can be mitigated by improving the EBL resist and etching process, as well as thermal ox-

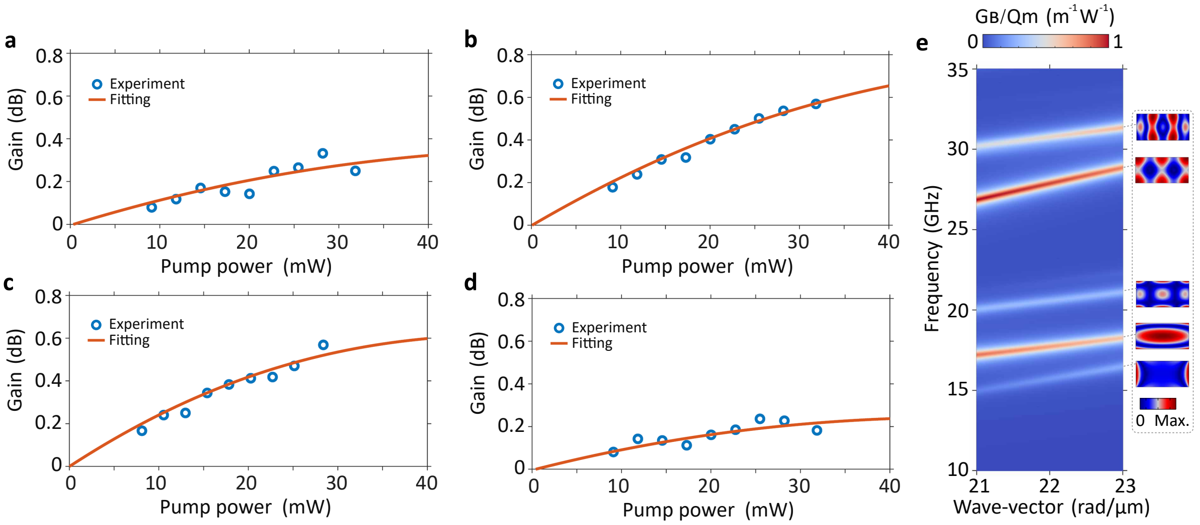

Supplementary Fig. 9. Fitting of backward Brillouin gain coefficient and acoustic dispersion. **a-d** Backward Brillouin gain coefficient fitting of SARAWs with different parameters, corresponding to the acoustic modes shown in Fig.4a-d in the main text. **e** The dispersion relation of the backward SBS acoustic modes. The displacement magnitude of the acoustic modes is shown in the gray dashed boxes.

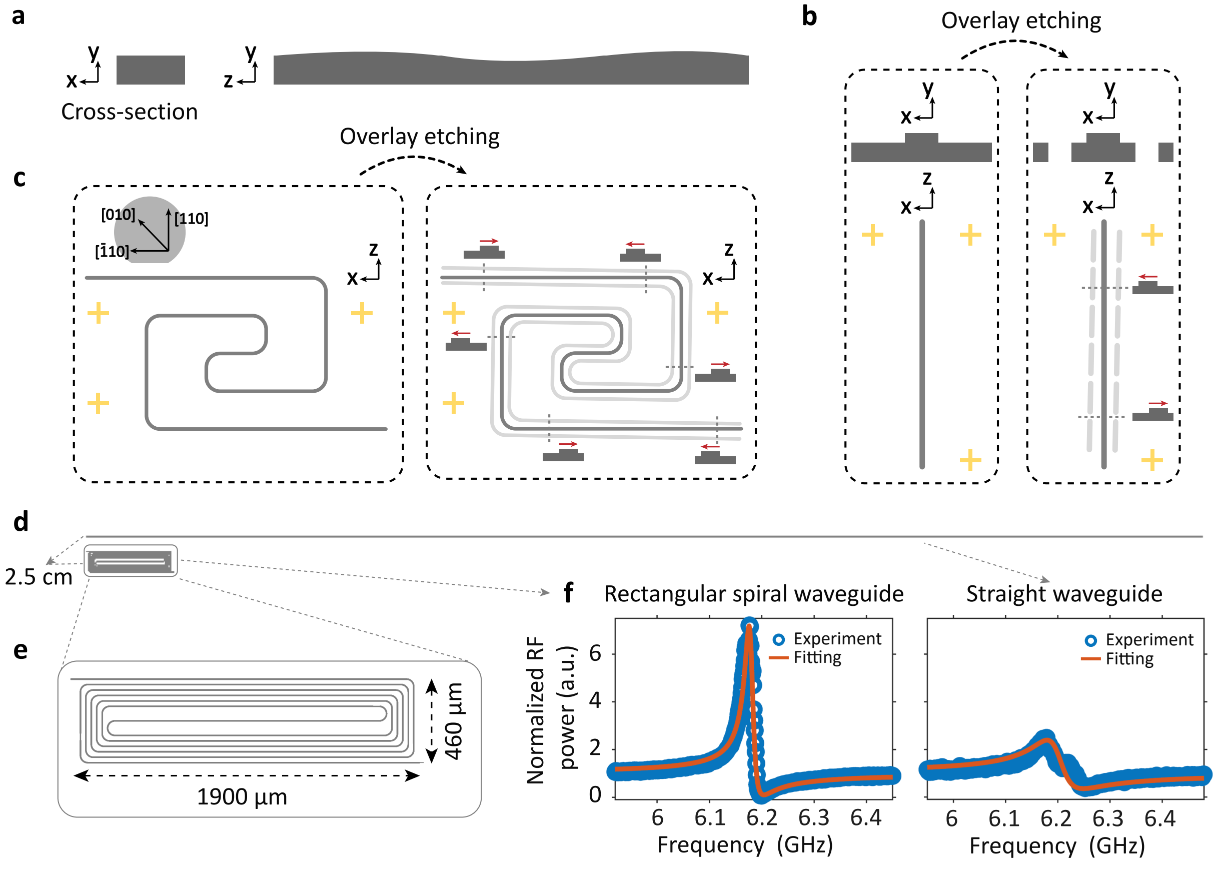

Supplementary Fig. 10. Inhomogeneous broadening and peak splitting. **a** The non-uniformity of silicon membrane. **b,c** The mismatches of straight and spiral waveguides induced by overlay exposure and etching. The golden crosses indicate the marks required for alignment, the dark grey lines denote rib waveguides, and the light grey lines represent the overlay etched regions. The upper left corner of the dashed box on the left side of figure **c** indicates the SOI wafer and the crystal orientations. **d,e** Schematics of the foot-prints of straight and rectangular spiral waveguides, and the enlarged view (**e**). **f** The heterodyne FWM experiment results of straight (right) and rectangular spiral (left) waveguides.

idation. For long-scale defects, further analysis is performed in this work. Since the straight waveguide layout (Supplementary Fig.10a) spans a longer distance, it is more susceptible to long-range fluctuations. The spiral waveguide with a smaller footprint is expected to show less inhomogeneous broadening. However, as reported in previous work [12], even more severe inhomogeneous broadening is observed in spiral waveguides compared to straight waveguides with the same length.

It implies that other factors induce inhomogeneous broadening in the spiral waveguide and take on a dominant role. The reasons for this phenomenon can be described as follows. For waveguide structures that require overlay exposure, such as suspended rib waveguides [1], alignment of the marks in overlay exposure introduces mismatches between the pattern layers (Supplementary Fig.10b). On our fabrication platform, the mismatches are larger than 20 nm, which causes drift in the waveguide cross-sectional geometry and results in inhomogeneous broadening. In particular, for spiral waveguides, this mismatch is dispersed throughout the spiral, leading to more severe inhomogeneous broadening and even peak splitting (Supplementary Fig.10c). However, SARAWs fabricated based on the loading-effect etching technique can avoid overlay exposure, thus eliminating the inhomogeneous broadening from alignment mismatches.

Moreover, the inhomogeneous broadening in spiral waveguides can also arise from the variation in crystal orientation. The eigenfrequencies of acoustic modes shift in different crystal orientations. In the case of spiral waveguides, such as Archimedean spirals, the waveguide direction shifts between [110] and [010] orientations. This variation in orientation leads to noticeable inhomogeneous broadening and peak splitting. To mitigate inhomogeneous broadening induced by crystal orientation, we opt for a rectangular spiral layout in SARAWs, which have the same crystal orientation in the orthogonal directions.

To prove the effectiveness of the method mentioned above, we fabricated 2.5-cm-long SARAWs with W_1 = 700 nm. We compare forward SBS results of the heterodyne FWM experiment measured in the rectangular spiral (G_B = 1,690 $W^{-1}m^{-1}$, Q_m = 325) and straight (G_B = 678 $W^{-1}m^{-1}$, Q_m = 90) SARAWs (Supplementary Fig.10f). Compared to straight SARAW, spiral SARAW exhibits not only a smaller footprint, but also an enhancement of 2.5 times in the gain coefficient and a 3.6 times increase in the mechanical quality factor.

[1] Kittlaus, E. A., Shin, H. & Rakich, P. T. Large Brillouin amplification in silicon. *Nature Photonics* **10**, 463–467 (2016).

[2] Gao, M. *et al.* Probing material absorption and optical nonlinearity of integrated photonic materials. *Nature Communications* **13**, 3323 (2022).

[3] Wilson, D. J. *et al.* Integrated gallium phosphide nonlinear photonics. *Nature Photonics* **14**, 57–62 (2020).

[4] Zhang, X. *et al.* Characterizing microring resonators using optical frequency domain reflectometry. *Optics letters* **46**, 2400–2403 (2021).

[5] Payne, F. P. & Lacey, J. P. A theoretical analysis of scattering loss from planar optical waveguides. *Optical and Quantum Electronics* **26**, 977–986 (1994).

[6] Afshar, S. & Monro, T. M. A full vectorial model for pulse propagation in emerging waveguides with subwavelength structures part i: Kerr nonlinearity. *Optics express* **17**, 2298–2318 (2009).

[7] Hon, N. K., Soref, R. & Jalali, B. The third-order nonlinear optical coefficients of si, ge, and si1- xgex in the midwave and longwave infrared. *Journal of Applied Physics* **110** (2011).

[8] Wiederhecker, G. S., Dainese, P. & Mayer Alegre, T. P. Brillouin optomechanics in nanophotonic structures. *APL photonics* **4** (2019).

[9] Schmidt, M. K., O'Brien, M. C., Steel, M. J. & Poulton, C. G. ARRAW: anti-resonant reflecting acoustic waveguides. *New Journal of Physics* **22**, 053011 (2020).

[10] Stern, I., Carosi, G., Sullivan, N. & Tanner, D. Avoided mode crossings in cylindrical microwave cavities. *Physical Review Applied* **12**, 044016 (2019).

[11] Gosalvez, M. A. *et al.* Simulation of microloading and arde in drie. In *2015 Transducers-2015 18th International Conference on Solid-State Sensors, Actuators and Microsystems (TRANSDUCERS)*, 1255–1258 (IEEE, 2015).

[12] Wang, K. *et al.* Demonstration of Forward Brillouin Gain in a Hybrid Photonic–Phononic Silicon Waveguide. *ACS Photonics* **8**, 2755–2763 (2021).

[13] Afshar, S. & Monro, T. M. A full vectorial model for pulse propagation in emerging waveguides with subwavelength structures part i: Kerr nonlinearity. *Optics express* **17**, 2298–2318 (2009).

[14] Shin, H. *et al.* Tailorable stimulated brillouin scattering in nanoscale silicon waveguides. *Nature communications* **4**, 1–10 (2013).

[15] Van Laer, R., Kuyken, B., Van Thourhout, D. & Baets, R. Interaction between light and highly confined hypersound in a silicon photonic nanowire. *Nature Photonics* **9**, 199–203 (2015).

[16] Van Laer, R., Bazin, A., Kuyken, B., Baets, R. & Van Thourhout, D. Net on-chip brillouin gain based on suspended silicon nanowires. *New Journal of Physics* **17**, 115005 (2015).

[17] Eggleton, B. J., Poulton, C. G., Rakich, P. T., Steel, M. J. & Bahl, G. Brillouin integrated photonics. *Nature Photonics* **13**, 664–677 (2019).

[18] Zurita, R. O., Wiederhecker, G. S. & Alegre, T. P. M. Designing of strongly confined short-wave Brillouin phonons in silicon waveguide periodic lattices. *Optics Express* **29**, 1736–1748 (2021).
